# 572. Assessing the Utility of Pan-scanning Among Burn Patients in the Emergency Department

**DOI:** 10.1093/jbcr/irag033.312

**Published:** 2026-04-06

**Authors:** Dharani Rao, Rachelle J Lodescar, Kerri Finnan, Megan E Molnar, Catherine Toal

**Affiliations:** Jerome L. Finkelstein, MD, Regional Burn Center, New Windsor, NY; Westchester Medical Center, Valhalla, , NY; Westchester Medical Center, Valhalla, , NY; Westchester Medical Center, Tarrytown, NY; Burn Center at Westchester Medical Center, White Plains, NY

## Abstract

**Introduction:**

Massive burn patients often undergo a full trauma work-up, including CT of the head, neck, chest, abdomen, pelvis commonly referred to as a “pan-scan.” However, burn patients are not always poly-trauma patients and this standardized approach fails to optimize the balance between the potential benefits of a scan with costs, radiation exposure, and delays in burn care. At many trauma centers, a patient with a massive burn automatically triggers a level 1 trauma activation in which patients undergo pan-scanning. However, more research is required to investigate the benefit of this approach. For this reason, we aim to investigate the utility of pan-scanning for identifying acute traumatic injury in patients with >20% TBSA burns at a level 1 trauma center with American Burn Association verification.

**Methods:**

We retrospectively analyzed narrative summaries of burn mechanisms, and imaging findings among 127 burn patients. Patient inclusion criteria was as follows; all patients admitted with >20% TBSA burns between 2015 and April 2025. Patients underwent analysis for the presence of traumatic injury following pan-scanning upon arrival. Pan-scanning was defined as receiving a CT head, neck/C-spine, and chest/abdomen/pelvis.

**Results:**

Among the 130 studied patients 22 were found to have been pan-scanned. All scanned patients regardless of findings presented with flame burns (i.e., vs. chemical, scald, or electrical). Of the 22 pan-scanned patients 10 were found to have traumatic injury. The pan-scanned patients who were not found to have traumatic injury presented with mixed mechanisms of trauma/burn; 3 explosions, 1 MVC, 4 house fires (1 possible fall, 2 found unresponsive, 1 with injury from falling structural components), 2 burns from gasoline ignition, 2 flash flame burns. Those patients in whom traumatic injuries were found were burned via the following mechanisms; 8 explosions, 1 found unresponsive in a house fire, 1 self-immolation with gasoline. The identified injuries included spinal fractures, rib fractures, subdural hematoma, lung contusions/hemorrhage, and craniofacial fractures. No identified injuries indicated immediate or operative intervention.

**Conclusions:**

Among pan-scanned patients less than half had traumatic injury. Our findings suggest that pan-scanning may not prove to have significant benefit specifically in situations where there is no evidence of traumatic mechanism despite level 1 trauma activation. Future studies are required for further optimization of indications for pan-scanning in the initial assessment of burn patients.

**Applicability of Research to Practice:**

CT pan-scanning in the initial assessment of massive burn patients is a useful tool for identifying acute traumatic injury but has questionable overall benefit when considering delays in the care of large burns.

**Funding for the study:**

N/A.

